# Management of adult focal nasolacrimal duct stenosis: long-term outcomes of 3D CT-DCG-assisted and endoscopically guided coronary catheter balloon dacryoplasty

**DOI:** 10.1038/s41598-024-66354-w

**Published:** 2024-09-30

**Authors:** Izabela Nowak-Gospodarowicz, Rafał Nowak, Michal Kinasz, Aleksandra Kinga Kicinska, Marek Rękas, Mohammad Javed Ali

**Affiliations:** 1 Department of Ophthalmology, Military Institute of Medicine-National Research Institute, 128 Szaserow St., 04-141 Warsaw, Poland; 2 Department of Ophthalmology, Jozef Strus City Hospital, Poznan, Poland; 3 Govindram Seksaria Institute of Dacryology, L.V. Prasad Eye Institute, Hyderabad, India

**Keywords:** Epiphora, Nasolacrimal duct stenosis, Nasolacrimal duct obstruction, Coronary balloon dacryoplasty, Lacrimal, Endoscopic lacrimal surgery, Diseases, Medical research

## Abstract

The purpose was to evaluate the use of 3D CT-DCG-assisted and endoscopically guided coronary catheter balloon dacryoplasty (CC-BDCP) in adults with focal stenosis of the nasolacrimal duct (NLD) and report their long-term outcomes. A prospective, non-randomized, single-center clinical study was performed, and the patients underwent endoscopy-guided CC-BDCP using percutaneous transluminal coronary angioplasty (PTCA) balloon catheters. 25 patients were enrolled in the study. The CC-BDCP procedure was performed in 21 of 25 (84%) patients, and the remaining 4 (16%) patients had significant procedural difficulties due to unfavorable anatomical conditions. Of the 21 patients, 10 (47.6%) were procedurally assessed as “easy” (eCC-BDCP) and in 11 (52.4%) as procedurally “difficult” (dCC-BDCP). Values on Munk's epiphora intensity scale changed overall from 4.0 preoperatively to 1.4 ± 1.6 (*p* = 0.00001) postoperatively overall. FDDT changed overall from 2.9 ± 0.3 to 1.1 ± 1.2 after treatment (*p* = 0.00008) (from 2.8 ± 0.4 to 0.3 ± 0.6 in the eCC-BDCP group and from 2.9 ± 0.3 to 1.4 ± 1.2 in the dCC-BDCP group (*p* = 0.01352). The anatomical and functional success rate was 77% overall, 90% in the eCC-BDCP group, and 64% in the dCC-BDCP group. The CC-BDCP led to a statistically significant decrease in epiphora in a particular group of adult patients with demonstrable focal stenosis of the NLD.

## Introduction

Epiphora accounts for about 3% of all complaints reported to ophthalmologists^1–3^. The most common cause in adults is primary, acquired partial, or complete nasolacrimal duct obstruction (PANDO)^4,5^. Focal stenosis can affect any segment of the lacrimal drainage system, leading to epiphora, impaired vision, and reduced patient quality of life^6,7^. Occasionally this may predispose the patient to developing acute inflammation of the lacrimal sac. Symptomatic obstruction of the nasolacrimal duct (NLD) in adults are usually managed by performing a dacryocystorhinostomy (DCR) using an external or trans-nasal endoscopic approach^4,8^. Reports on the effectiveness of these methods are characterized by a high success rate, ranging from 70 to 97%^4,9,10^. These procedures lead to irreversible changes in the lacrimal drainage anatomy, are usually performed under general anesthesia, and carry the risk of complications, as with many invasive procedures. In the era of minimally invasive lacrimal surgery, it is not unusual to visit or revisit lesser invasive yet equally effective management options with a preferable preservation of the natural lacrimal drainage anatomy.

Balloon dacryoplasty (DCP) was first described in 1989 by Becker and Berry as non-incisional dilatation of the nasolacrimal duct^11^. This technique, with further modifications, gained popularity in treating congenital nasolacrimal duct obstruction (CNLDO), with a reported success rate ranging from 76 to 83%^12–15^. The efficacy of this procedure in adults remains to be determined. The literature shows that the effectiveness of BDCP in PANDO ranges from 11.1 to 100%^16–20^. Due to the high cost of lacrimal catheters (LacriCATH by Quest Medical Inc, USA, and Ophtacath by FCI, France), the use of percutaneous transluminal coronary angioplasty (PTCA) balloon catheters was described by some authors as a cost-effective alternative^21,22^. Studies on the treatment of partial nasolacrimal duct obstruction in adults are few, usually retrospective, with heterogeneous inclusion and success criteria, based on small groups of patients, or short follow-up^11,23–25^. The present study aimed to evaluate the long-term outcomes with the use of 3D CT-DCG-assisted and endoscopy-guided coronary catheter balloon dacryoplasty (CC-BDCP) in symptomatic patients with focal stenosis of the nasolacrimal duct presenting clinically as partial NLDO.

## Material and methods

### Study design

The study followed the Tenets of the Declaration of Helsinki and was approved by the bioethics committee at the Military Medical Chamber. A prospective, non-randomized, single-center clinical study was performed spread across two years (2021 and 2022). The study was conducted at the tertiary Ophthalmology Care unit at the Military Institute of Medicine- National Research Institute, Warsaw, Poland.

### Study groups

The study group included adult males and females whose health conditions warranted participation for at least 12 months. Eligible patients acknowledged their understanding and ability to follow the study requirements. Each patient signed a written informed consent form for the treatment and participation in the study.

Each patient met the inclusion criteria: (1) age over 18, (2) one or both eyes affected by epiphora, and (3) clinically confirmed partial nasolacrimal duct obstruction and radiologically confirmed focal stenosis.

The exclusion criteria were (1) age under 18 years; with (2) no significant epiphora; (3) diagnosis of complete NLDO; (4) history of acute inflammation of any part of the lacrimal tract, nasal cavity, or paranasal sinuses; (5) significant abnormalities of the eyelids; (6) significant nasal abnormalities upon endoscopic examination; and (7) history of past surgical interventions on the lacrimal drainage apparatus.

A detailed medical history was taken preoperatively with special attention to the duration of symptoms and triggers of epiphora, history of inflammation or infection, past injuries or surgical interventions, concomitant allergies, drug allergies, and previous medical treatment and its effectiveness.

**Figure 1:**
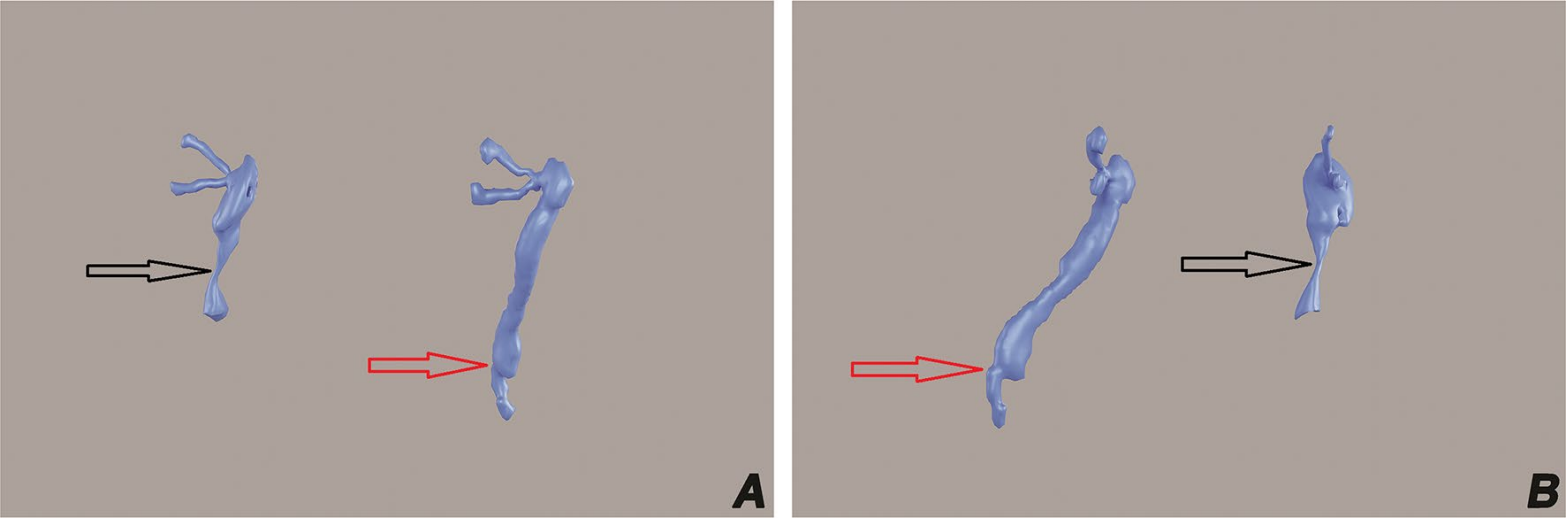
CT-DCG reconstruction: Three-dimensional reconstructions of a CT-DCG showing anterior view (Panel **A**) and lateral view (Panel **B**). Note two lacrimal sacs, one of them in each panel shows a gross narrowing at the middle of the nasolacrimal duct (black arrow, Panels **A** and **B**). This narrowing is smooth without change of angulation and would be easier amenable to BDCP. The other lacrimal sac shows abrupt change of angulation following the stenosis (red arrow, panels **A** and **B**) and proximal dilatation. These cases would be difficult technically during a BDCP procedure.

The intensity of epiphora was assessed based on the Munk scale: from 0 to 4 (Munk et al., 1990). The fluorescein dye disappearance test (FDDT) was performed and graded from 0 to 3 in every patient in the standard fashion^26–28^. The preoperative assessment included best-corrected visual acuity (BCVA) and slit lamp ophthalmological examination with fundus examination. An ocular examination was performed to exclude other causes of epiphora, such as malposition of the eyelids, blepharitis, conjunctivitis, narrowing, and atresia of lacrimal puncta, canaliculitis, abnormal growth of eyelashes, dry eye syndrome, keratitis, lacrimal pump failure or overstimulation of the lacrimal gland. Nasal endoscopy was performed with a detailed evaluation of the nasal mucosa and nasal cavity anatomy. Irrigation and probing of the lacrimal drainage were performed to identify the level of obstruction. To visualize the location of the stenosis and assess the anatomy of NLD and nearby structures, a 3D computed tomography-dacryocystography (3D CT-DCG) was performed and reconstructed to have greater details (Fig. 1A,B).

Patients with clinically diagnosed partial PANDO and radiologically confirmed focal stenosis of the nasolacrimal ducts underwent coronary catheter balloon dacryoplasty (CC-BDCP) using a PTCA catheter (Boston Scientific NCEmerge PTCA Dilatation Catheter, balloon 3 × 15 mm, Boston Scientific, MA, USA), followed by intubation of the lacrimal tract with a Masterka stent (FCI Ophthalmic, Paris, France). NC EMERGE™ is a disposable, noncompliant balloon with a hydrophilic coating used in cardiology for coronary angioplasty. It needs a guide wire to facilitate insertion in the stenosed segment. The catheter requires an inflation device that inflates it with liquid to the desired pressure.

### Surgical technique (Fig. 2)

**Figure 2:**
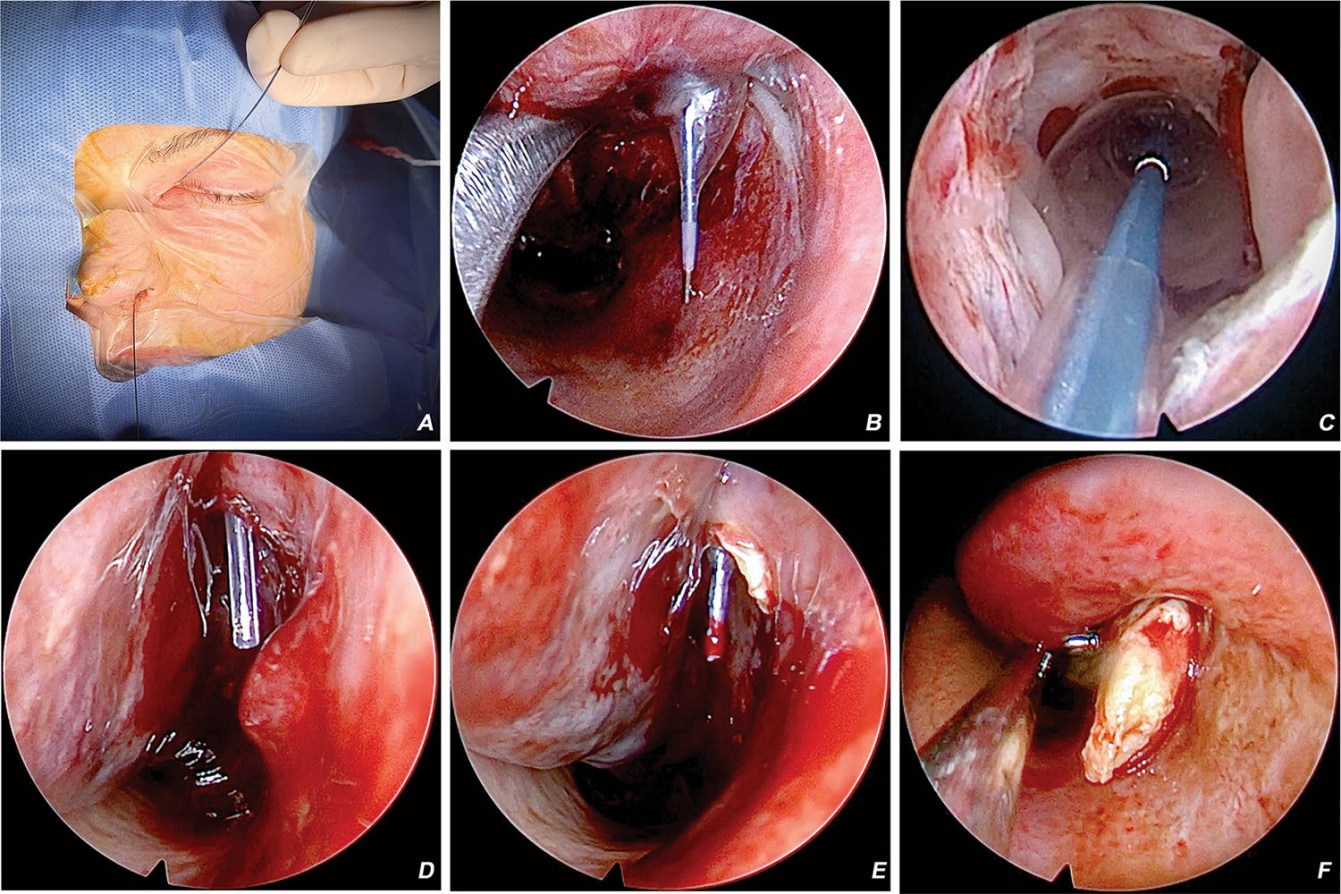
CC-BDCP procedure. Clinical image showing balloon canulation of the lacrimal system (Panel A). Endoscopic image showing balloon dilatation of the NLD (view in the inferior meatus—panel B) (end on NLD interior view—panel C). Endoscopy image showing Masterka^R^ stent in-situ (panel D). Endoscopy view of a difficult case which reveals a NLD dacryolith soon after BDCP (panel E), which was then extracted from the NLD (panel F).

All procedures were conducted under general anesthesia. Visualization of the nasal cavity was ascertained with a 4 mm 0-degree endoscope. Before surgery the nasal cavity was packed with sponges soaked in adrenaline and lignocaine solution (1:10,000). After decongestion, the inferior turbinate was medialized with a freer elevator. The NLD was gently probed subsequently with a 23-gauge lacrimal probe cannula (size 0.64 × 60 mm, Geuder, Heidelberg, Germany) under endoscopy guidance. Then, a Ritleng probe (FCI, France) was inserted in the lacrimal tract with visualizing its tip in the inferior meatus. The balloon guide wire was inserted through the Ritleng probe to the floor of the nasal cavity. The probe was then removed, and the guide wire stayed in place. A 3 × 15 mm catheter was beaded on the guide wire and inserted into the NLD until it reached the inferior meatus. In the place of the obstruction, the balloon was filled with saline using an inflator up to 8 atm for 90 s and then for 60 s. After balloon deflation, the guide wire was removed first, followed by the catheter. A Masterka^R^ stent was inserted into the NLD. The lacrimal drainage was irrigated with a topical steroid (Depo-Medrol), and the patency was simultaneously confirmed. The inferior turbinate was gently repositioned.

Amongst the 21 patients where CC_BDCP was successfully carried out, the procedure was assessed as easy in 10 patients (47.6%) (hereafter the eCC-BDCP group), and in 11 (52.4%) was assessed as “difficult” (the dCC-BDCP group). Difficult CC-BDCP was defined when there were technical difficulties in performing the procedure, prolonged duration, bleeding, and requiring multiple maneuvers to position the balloon segment within the stenotic area (Figs. 1 and 2).

The postoperative regime consisted of antibiotic-steroid eyedrops for four weeks. Nasal mometasone spray (mometasone furoate; 50 µg/dose) was administered as a spray for two weeks. The Masterka stent was extubated four weeks after the procedure.

Postoperative visits were scheduled for the first day, then 1st, 3rd, 6th, and 12th months after surgery. In case of complications, additional visits were indicated at each examination period. The final outcomes were considered at the end of a 1-year postoperative period. The success of the procedure was assessed subjectively on the Munk scale and objectively by lacrimal drainage irrigation and change in the FDDT before and after surgery.

### Statistical analysis

Statistical analysis was performed using SPSS software (IBM Corp. Released 2012. IBM SPSS Statistics for Windows, Version 21.0. Armonk, NY: IBM Corp, USA). The normality of the distribution was measured by the Shapiro–Wilk test. Survey data were presented in a descriptive form. The Wilcoxon pair order test was used to compare the two dependent groups. The Mann–Whitney U test was used to compare the two independent groups. A significance level of *p* < 0.05 was considered significant.

## Results

Twenty-five patients (14 females and 11 males) aged 57 ± 19 years (range 19–81) were included in this study. There was no difference in the laterality (12 right eye and 13 left eye). The CC-BDCP procedure was performed in 21 of 25 (84%) patients, and the remaining 4 (16%) patients had significant procedural difficulties due to unfavorable anatomical conditions, such as deep orbit and long NLD with many changes of axis angles (Fig. 1).

Nineteen of 21 (91%) patients were intubated with a Masterka stent (FCI Ophthalmics, Paris, France). Two patients (9.5%) were not intubated due to the intraoperatively recognized presence of associated dacryoliths proximal to the stenotic segment with associated inflammation (Fig. 2E,F). All stents were extubated at four weeks, and a minimum follow-up of 12 months was considered for final analysis.

**Figure 3:**
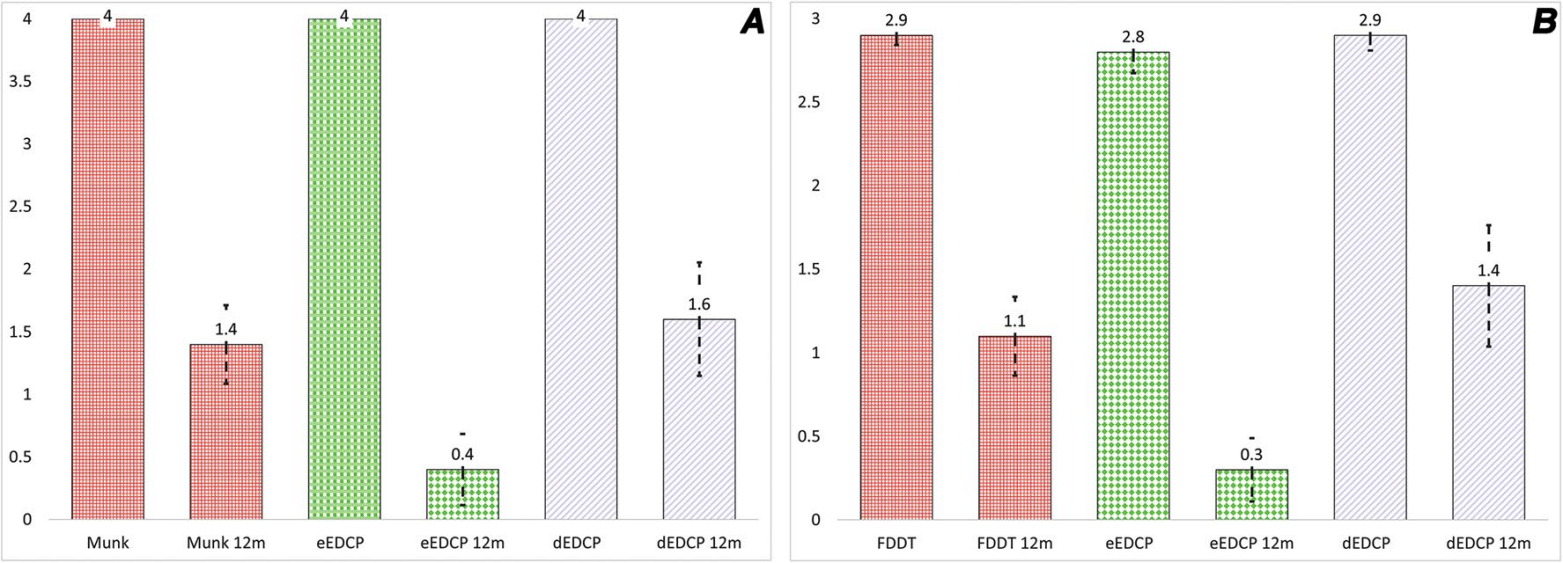
CC-BDCP outcomes: Bar plot showing outcomes at 12- months follow up in the Munk’s scale recordings (Panel **A**) and Fluorescien dye disappearance test [FDDT] (Panel **B**). The tall columns represent the preoperative score and the smaller columns beside represent postoperative score. The red columns represent overall change in the score, whereas the green columns represent the eBDCP group and the grey columns the dBDCP group.

Value changes on Munk's epiphora intensity scale were statistically significant, from 4.0 to 1.4 ± 1.6 at the 12-month follow-up for all patients (*p* = 000001, Wilcoxon signed rank test), from 4.0 to 0.4 ± 0.9 in the eCC-BDCP group, and from 4.0 to 1.6 ± 1.5 in the dCC-BDCP group (*p* = 0.01596, Mann–Whitney U Test (Fig. 3A).

The FDDT score change was statistically significant from 2.9 ± 0.3 to 1.1 ± 1.2 after treatment (*p* = 0.00008, Wilcoxon signed rank test) for all patients, from 2.8 ± 0.4 to 0.3 ± 0.6 in the eCC-BDCP group and from 2.9 ± 0.3 to 1.4 ± 1.2 in the dCC-BDCP group (*p* = 0.01352, Mann–Whitney U Test, Fig. 3B). The overall anatomical and functional success rate was noted in 76% (16/21). The success rate analyzed by groups was noted to be 90% in the eCC-BDCP group and 64% in the dCC-BDCP group. Five patients (24%) had a recurrence of epiphora between 4 and 9 months following the procedure. Follow-up CT-DCG in these patients showed recurrence of the stenosis in three and recurrence of the dacryoliths in two patients who were not earlier intubated. All these patients are planned for an endoscopic DCR. There were no technical issues with the balloons, inflation device, or stents.

## Discussion

Balloon dacryoplasty has been in practice for the last three decades as a minimally invasive alternative to several lacrimal drainage procedures. However, the data regarding its utility in adults with focal NLD stenosis remains unclear^20,29^. Inconsistent data on the procedure success rates vary from 36.67 to 90%^16–20,24^. The only repeatable finding from previously published reports was a recommendation to use this procedure for the treatment of partial NLD obstruction in adults^17–19,30^.

To assess the efficacy of coronary catheter BDCP with its several advantages over the traditional BDCP catheters^22^, the present study embarked on to study its benefits in patients with demonstrable focal NLD stenosis. Of the 21 patients that were used for the final analysis, it was surprising that nearly half of them were placed in the difficult group due to procedural difficulties in accurately reaching the focal stenotic segment, anatomical variations, exaggerated angle between the lacrimal sac and the nasolacrimal duct, multiple angulations within the NLD, change of direction in the distal-most segment near the inferior meatal opening, deep sockets, long nose, and lower than usual location of the meatal opening. Endoscopic guidance helped the surgeons from uncontrolled pushing of the balloon probe, false passage, and damage to the NLD, which may not be noticed without endoscopy guidance. These subgroups of patients were technically challenging, despite using the anterograde technique and contrary to previous reports, where a procedural failure because of the inability to catheterize the NLD had been reported only with the use of the retrograde technique^29,31–33^.

The overall anatomical and functional success rate of 77% in this study was significantly higher in the group in which the procedure was easy to perform (eCC-BDCP – 90%) compared to the group in which procedural difficulties were noted (dCC-BDCP – 64%). This suggests that in a select group of easy patients, the efficacy of the procedure may have similar outcomes to those with a more invasive DCR-external or endoscopic. Delaney and Khooshabeh report an 84% success rate of external DCR for adult patients with partial NLD obstruction at the four-month follow-up and 70% at the three-year follow-up^9^. Wormald and Tsirbas report an 84% success rate of endonasal DCR in adult patients with partial NLD obstruction at a minimum of 12 months follow-up^10^. To compare, the overall long-term success rate of balloon dacryoplasty for partial distal NLDO reported by Konuk et al. at the mean 100 ± 38-month follow-up was 73.3%^19^ and was similar to the rates reported in other studies^21,34,35^. Improvement in epiphora in 90% of 142 patients from the largest study on balloon dacryoplasty may confirm this assumption^17^.

Limitations of this study include a small sample size, which was further divided postoperatively into two smaller groups, and a lack of dacryoendoscopic assessment. The other concerns could be higher radiation exposure (CTDCG) in this subset of patients and the need for general anesthesia. However, its strengths are uniform long-term assessment, 3D CT-DCG and endoscopy guidance, and the use of CC-BDCP. The procedure in some adults may be technically challenging and sometimes impossible to perform because of unfavorable anatomical conditions, especially in cases with acute angulation within different segments of the nasolacrimal duct and very low placement of the NLD opening into the inferior meatus. A thorough examination of the patient supported by computed tomography imaging allows for assessing anatomical conditions and proper qualification for this procedure. In most cases, the procedure performed with diligence under endoscopic guidance does not cause complications.

## Conclusions

Endoscopy-guided and 3D CT-DCG-assisted coronary catheter balloon dacryoplasty led to a statistically significant decrease of epiphora in a particular subset group of adult patients with clinically partial nasolacrimal duct obstruction. A further prospective study is needed to assess the effectiveness of the 3D CT-DCG imaging to predict several anatomical factors and the difficulty level one could anticipate prior to CC-BDCP and formulate appropriate inclusion criteria for an effective endoscopy-guided balloon dacryoplasty in adults.

## Data Availability

All data generated or analysed during this study are included in this published article.
